# Inferior Part of Rectus Abdominis Muscle Flap Outcomes after Abdominoperineal Resection: A Case Series Pilot Study

**DOI:** 10.29252/wjps.10.3.104

**Published:** 2021-09

**Authors:** Mahdi Alemrajabi, Morteza Khavanin Zadeh, Nima Hemmati, Behrouz Banivaheb, Fatemeh Alemrajabi, Sepideh Jahanian, Mohammad Bahadoram, Maedeh Barahman

**Affiliations:** 1 Firoozgar Clinical Research Devel-opment Center (FCRDC), Firoozgar hospital, Iran University of Medical Sciences (IUMS), Tehran, Iran.; 2 Hasheminejad Kidney Center (HKC), Iran University of Medical Sciences (IUMS), Tehran, Iran.

**Keywords:** Abdominoperineal resection, Rectaloanal malignancies, immediate flap reconstruction

## Abstract

**BACKGROUND:**

The standard surgical treatment for low rectal cancer is abdominoperineal resection (APR). Comparing to primary closure, immediate flap reconstruction has shown to have good outcomes. We aimed to assess the inferior rectus abdominis muscle flap complications after APR surgery, a new method of reconstruction.

**METHODS:**

This study was conducted from 2014 to 2016 in a single center in Firoozgar Hospital, Tehran, Iran. Eighteen patients who underwent pelvic floor closure with inferior part of abdominis rectus musculofascial flap were included enrolled. The sampling method used in this study was census. All patients had distal rectoanal malignancies. A checklist including age, gender, tumor location, complications after surgery, tumor type, length of hospital stay, length of operation, neoadjuvant chemotherapy and neoadjuvant radiotherapy history was filled for all patients.

**RESULTS:**

Among 18 participants, 27.8% were female. The mean age of participants was 58.28 ± 17.86 yr (minimum of 19 and the maximum of 89 yr). The pathology of the tumor in all but one of the cases was adenocarcinoma (94.4%). The overall complication rate after surgery was 27.8%. In total, 80% received neoadjuvant chemoradiotherapy. In 12 months follow-up 2 patients needed reoperation.

**CONCLUSION:**

Inferior part of rectus abdominis muscle flap was a reliable and comparable means of reconstruction after APR surgery with low rate of complications and mortality.

## INTRODUCTION

The standard surgical treatment for low rectal cancer is abdominoperineal resection (APR). Usually, extensive resections produce an extensive pelvic defect that present complications including wound closure challenges, infections and dehiscence. Comparing to primary closure, it had been shown that immediate flap reconstruction has fewer complications^1^^-^^6^. 

A large, non-collapsible dead space in pelvis is associated with wound-healing problems complications. Pelvis irridations also adds to the problem. Surgeons suggested different types of myocutaneous flap techniques to provide coverage for blank space in perineum^1^^, ^^2^^, ^^7^^, ^^8^. 

Myocutaneous flaps provide bulky and healthy tissues which are well vascularized to fill the empty space of perineum, thus improving better wound healing and decreased infection rates. It can also provide better access for immune system agents to the area and increase oxygenation^8^. 

Different options for pelvic reconstruction include pedicled vertical rectus abdominis myocutaneous (VRAM) flaps, local V to Y advancement flaps and pedicled gracilis muscle flaps. There are several pros and cons for the existing flaps. The pedicled VRAM flaps has fewer rate of complications than primary wound closure. A decrease of perineal morbidity has been also seen in VRAM flaps and it also had a well long term survival. Compared to gracilis flaps, VRAM flaps have shown better results^9^^-^^17^. VRAM flap method use is limited by some factors including previous abdominal surgery, positioning, scarring and etc^9^. 

V to Y advancement flaps make less abdominal wall morbidity comparing VRAM flaps and the flap size is less bulky in comparison^18^^,^^19^. Although gracilis flaps may not be applicable to patients with larger pelvic defects^20^. 

 There is still no consensus regarding the best option for pelvic space filling. Thus, we aimed to assess the inferior rectus abdominis muscle flap complications after APR surgery. 

## METHODS AND MATERIALS

Among 204 cases of proctectomy in 2014 to 2016, in Firoozgar Hospital, Iran University of Medical Sciences, Tehran, Iran; 38 underwent abdominoperineal resection and 18 pelvic floor closure with inferior part of rectus abdominis flap reconstruction. Inclusion criteria were as follow; all patients had distal recto-anal malignancies with sphincter involvement. Female patients should have had a prior history of hysterectomy. Prior to surgery, patients underwent colonoscopy and biopsy. A computed tomography (CT) scan was performed for all patients to assess lymph node involvement and distant metastasis. 

Exclusion criteria were; 1) prior abdominal surgery or abdominal wall defects, 2) distant metastasis and terminal cancer, 3) adjacent organ involvement requiring a wide resection, 4) no consent to take part in the study. Finally, 18 patients underwent pelvic floor closure with inferior part of abdominis rectus muscle flap.

A checklist including age, gender, tumor location, complications after surgery, tumor type, length of hospital stay, length of operation, neoadjuvant chemotherapy and neoadjuvant radiotherapy history was filled for all patients. Patients were followed for complications in one-year period after surgery. The complications were defined as; 1) dehiscence, dermal separation more than one third of the wound but without infection, 2) abscess, a purulent collection requiring drainage, 3) flap loss, necrosis of at least one third of flapped tissue, 4) infection, a mucopurulent discharge from the wound, 5) prolonged healing, absence of wound closure at 3 wk from surgery, 5) obstruction, nausea and vomiting with non-presenting feces in the colostomy with abdominal distension, 6) cellulitis, inflammation of the tissue around the wound requiring antibiotic treatment. 

***Surgical technique***

**Fig. 1:**
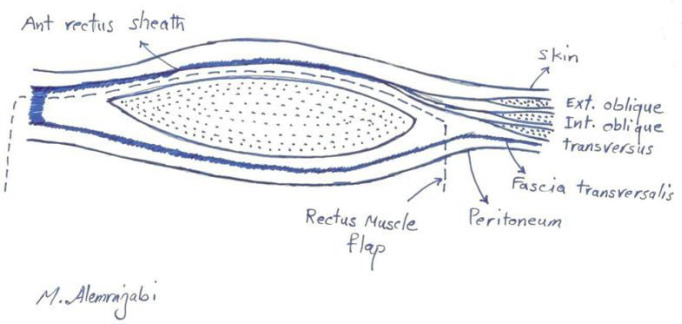
The skin and anterior fascia was dissected from the bulk of rectus abdominis muscle

**Fig. 2:**
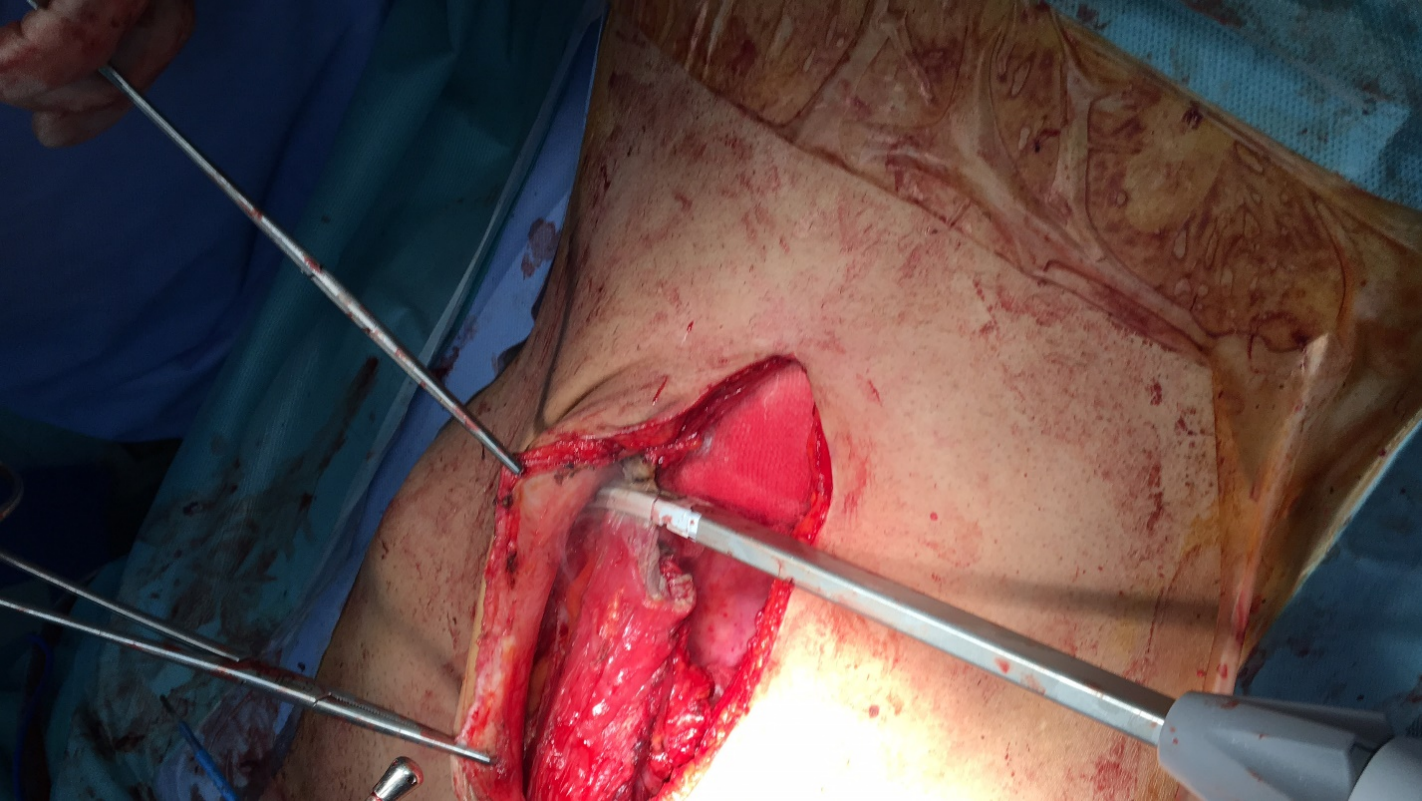
The inferior part of rectus abdominis muscle was cut just blow the arcuate line and medial to the semi lunar line

**Fig. 3:**
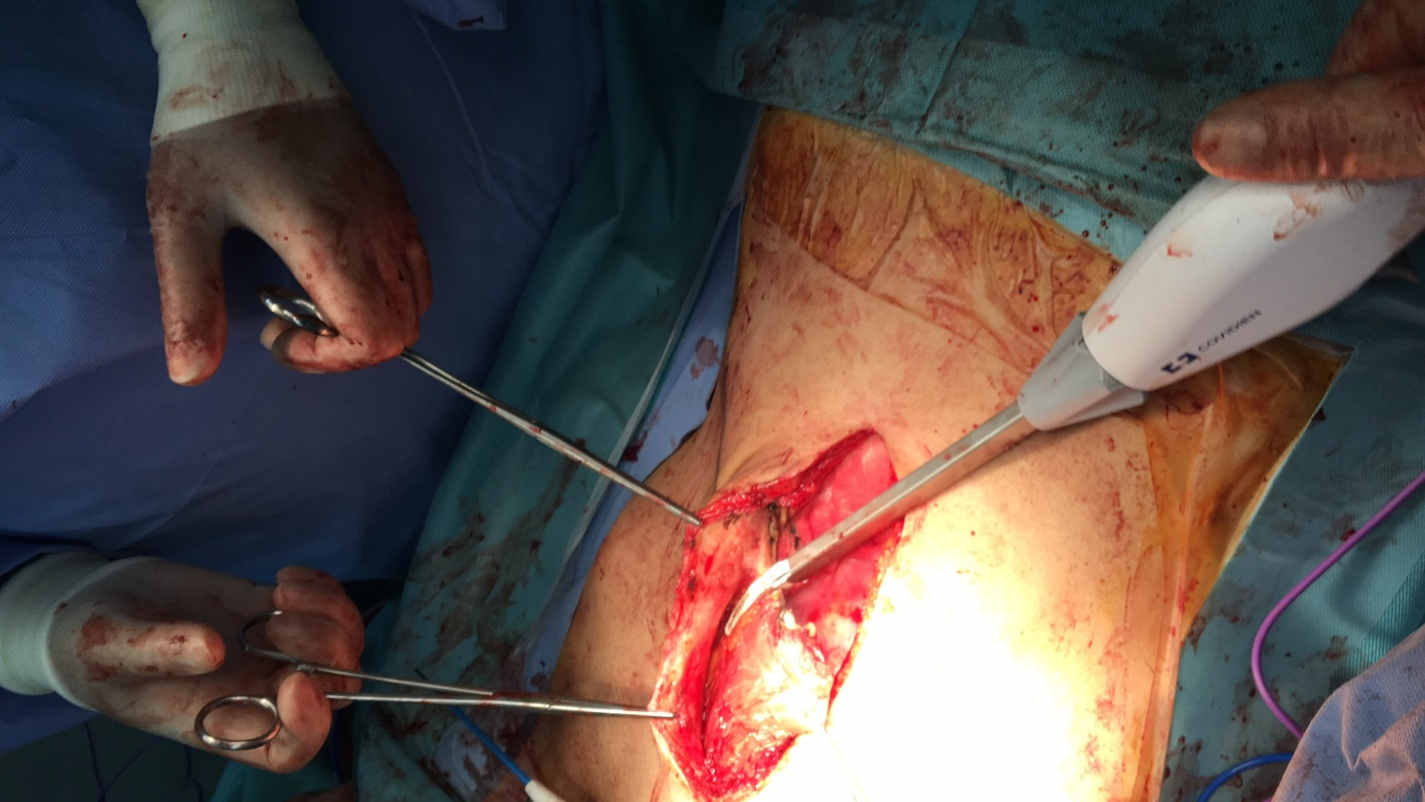
The inferior part of rectus abdominis muscle flap dissection was continued to the origin which isonthepubis

**Fig. 4:**
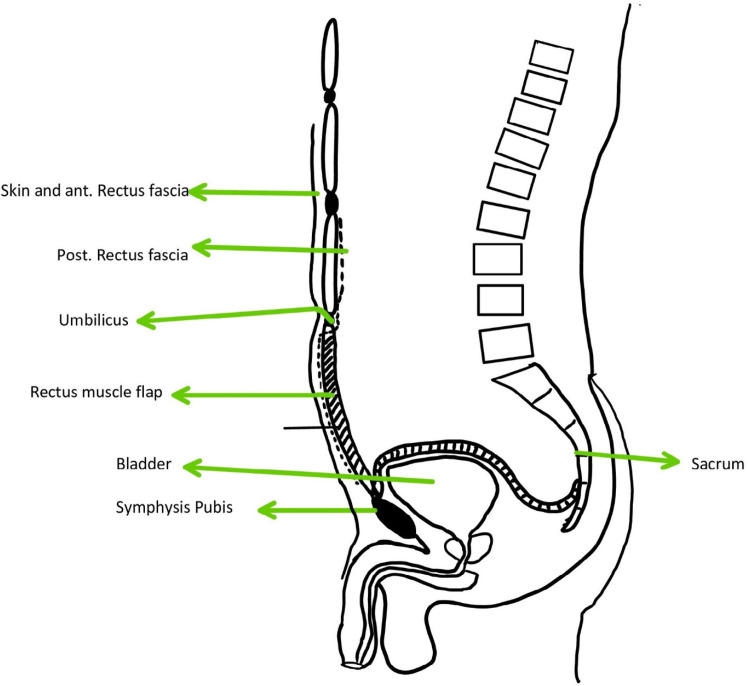
The inferior part of rectus abdominis flap was fashioned into the perineum

**Fig. 5:**
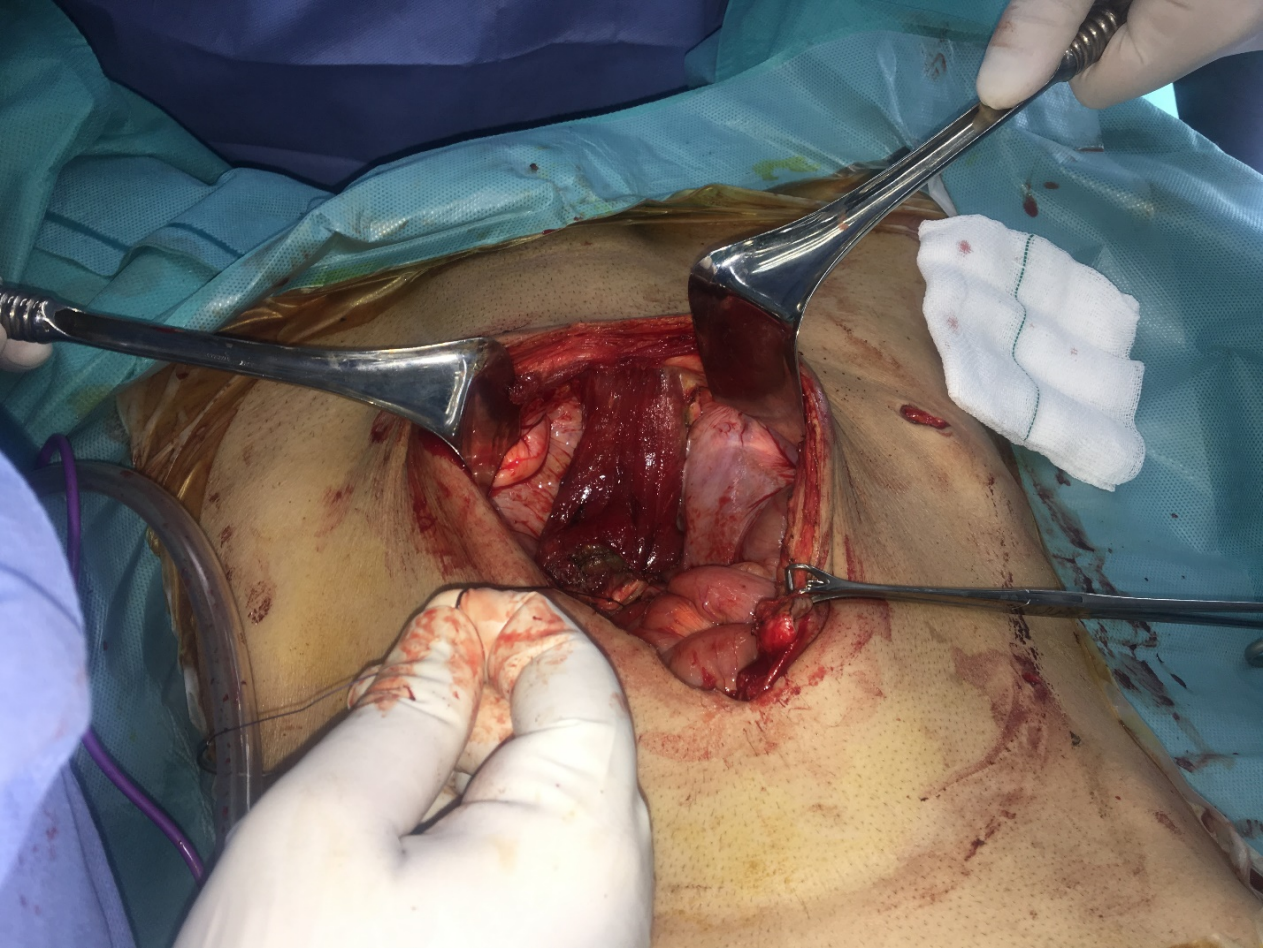
Multiple layered sutures were used to place the flap without tension

The method of reconstruction was Alem’s reconstruction method described in a previous case report^21^. In brief, thorough a midline incision, abdominal cavity was accessed. The inferior part of the right rectus muscle with its posterior fascia was dissected carefully down to its pubic origin (Figure 1). Appropreate location was selected to place colostomy on the left side (Figure 2). Maximum effort was made to preserve the perforating and inferior epigastric arteries (Figure 3). The flap was then placed in the pelvic vacant space and fixed with loose stiches (usually vicryl 3-0) without any tension (Figures 4 and 5). After placement of end colostomy, midline incision was closed using anterior rectus fascia of both sides and posterior fascia of the left side. Skin closed using 3-0 separate nylon stiches. 

***Statistical analysis***

For statistical analysis, IBM SPSS statistics 22 (IBM, Inc, New York, USA) was used. For numerical variables means ± standard deviations and for categorical variables frequencies were reported. 

***Ethical Approval***

The study protocol was approved by the Ethics Committee of Iran University of Medical Sciences (Ethical Code: IR.IUMS.REC.1396.921124). An informed consent was obtained from all participants and they were assured regarding data confidentiality. Patients were free to leave the study at any point without affecting their routine care. Helsinki declaration was obeyed in all stages.

## RESULTS

Among 18 participants, 27.8% were female. The mean age of participants was 58.28 ± 17.86 yr (minimum of 19 and the maximum of 89 yr). All patients had distal rectal cancer with sphincter involvement (T4a). The pathology of the tumor in all but one of the cases was adenocarcinoma (94.4%). No patient had distant metastasis. All of the patients received neo-adjuvant chemoradiotherapy. 

The mean duration for surgery was 3.30 ± 0.54 h (minimum of 2 and the maximum of 4 h). The mean hospital stay after surgery was 6.55 ± 3.07 d (minimum of 3 and the maximum of 17 days). It took 1.11 ± 0.22 d for patients to get out of bed. The mean duration to start oral nutrition was 2.05 ± 0.8 days. A patient was hospitalized for 17 d to undergo irrigation from the APR defect every other day due to perforated distal rectal cancer.

Post-op complications are listed in Table 1. No flap loss, cellulitis, and prolonged healing was observed. Of these 18 patients, 2 (11.1 %) needed a reoperation after complications (dehiscency, and obstruction). One patient with abscess formation underwent a radiologic drainage and the one with abdominal wound infection was treated conservatively with antibiotics and wound care. In the post-operative period, no mortality was observed. No patient developed deep vein thrombosis or pulmonary emboli, parastomal hernias or urinary tract infection. The frequency of abdominal hernia was nil. The patients were followed for 12 months at 1, 3, 6 and 12 months after discharge. Almost all patients complained about the colostomy and how it affected their quality of life but no questionnaire was filled. In one-month period after surgery no complications were seen considering the surgery. At 3 months post-op visit, no complications reported. However, at six visit and 12 months visit, 2 patients (11.2%) developed a nonfunctioning colostomy and went for reoperation. 

## DISCUSSION

Overall complication rate after surgery was 22.4%, among these patients 80% had received radiotherapy before surgery. No complications were observed for 6 months after surgery, but within 6 to 12 months, 2 patients developed non-functioning colostomy and were re-operated. No mortality was recorded in 12 months follow up after surgery. 

Flap reconstruction seems to be a preferred method of reconstruction in variety of surgeries^22^^,^^23^ and primary closure might be associated with some complications such as infection or dehiscence^24^. 

Sheckter et al. compared three different methods for pelvic floor reconstruction. The rate of complications is higher in V-to-Y advancement group (73.03%). However, VRAM flap was superior to the other two techniques (rate of complications; 22.2%). Due to limited sample size in the stated study, a rationale conclusion might not be possible^25^. 

Other investigations also verified The VRAM flap safety for abdominopelvic resection with complications up to about 10%. Considering the stage of malignancy, the complication rate could be varied. The overall complication rate of VRAM flaps were 12.96% to 22.86% in different stages of rectal cancers^26^. The complication rate in this study which required intervention was 15.2% after surgery. No patient had flap related complications. 

Moreover, no mortality was recorded in the hospital after surgery. This is in line with other studies that evaluated the outcomes of VRAM flaps after APR, and no mortality was reported^27^. Considering that no mortality was recorded, the inferior part of rectus abdominis muscle flap technique has a low mortality rate and comparable to other techniques of reconstruction^28^. 

Furthermore, dissection is less in the inferior part of rectus abdominis compared to VRAM flaps, therefore, post-op morbidities, especially abdominal wall ones are less common. Despite the fact, ostomy placement in VRAM is a matter of challenge but the left side is intact in rectus flap^9^^,^^25^. However, the muscular bulk in the rectus flap is less compared to VRAM, but it filled the pelvis appropriately. Further studies are recommended to investigate and compare the inferior rectus portion with the VRAM flap method in larger prospective studies.

We had some limitations; 1) the sample size of this study was low, so the results of this study cannot be generalized, 2) since it was a cross sectional study the preliminary results cannot be generalized so it is suggested to perform further studies with prospective design 3) the follow up period of this study should be extended. 

**Table 1:** The frequency of complications after abdominopelvic resection surgery with inferior part of rectus abdominis muscle flap and before discharge

Complications	Frequency	Percent
No complications	13	77.8
Wound infection	1	5.6
Dehiscence	1	5.6
Obstruction	1	5.6
Intra-abdominal abscess	1	5.6

## CONCLUSION

The inferior part of rectus abdominis muscle flap seems to be a reliable and comparable means of reconstruction after APR surgery with low rate of complications and mortality. 

## CONFLICT OF INTEREST

The authors disclose no conflict of interest.
